# The Role of Focal Adhesion Kinase in Keratinocyte Fibrogenic Gene Expression

**DOI:** 10.3390/ijms18091915

**Published:** 2017-09-07

**Authors:** Michael Januszyk, Sun Hyung Kwon, Victor W. Wong, Jagannath Padmanabhan, Zeshaan N. Maan, Alexander J. Whittam, Melanie R. Major, Geoffrey C. Gurtner

**Affiliations:** 1 Hagey Laboratory, Division of Plastic Surgery, Department of Surgery, Stanford University School of Medicine, Stanford, CA 94305-5148, USA; mjanuszyk@mednet.ucla.edu (M.J.); kwonsunh@stanford.edu (S.H.K.); vicw.wong@gmail.com (V.W.W.); jaganpa@stanford.edu (J.P.); zmaan@stanford.edu (Z.N.M.); alexander.whittam@gmail.com (A.J.W.); melaniermajor@gmail.com (M.R.M.); 2 Program in Biomedical Informatics, Stanford University School of Medicine, Stanford, CA 94305-5148, USA

**Keywords:** focal adhesion kinase, keratinocyte, mechanotransduction, extracellular matrix, single-cell transcriptional analysis, skin fibrosis, hypertrophic scar, transcriptomics

## Abstract

Abnormal skin scarring causes functional impairment, psychological stress, and high socioeconomic cost. Evidence shows that altered mechanotransduction pathways have been linked to both inflammation and fibrosis, and that focal adhesion kinase (FAK) is a key mediator of these processes. We investigated the importance of keratinocyte FAK at the single cell level in key fibrogenic pathways critical for scar formation. Keratinocytes were isolated from wildtype and keratinocyte-specific FAK-deleted mice, cultured, and sorted into single cells. Keratinocytes were evaluated using a microfluidic-based platform for high-resolution transcriptional analysis. Partitive clustering, gene enrichment analysis, and network modeling were applied to characterize the significance of FAK on regulating keratinocyte subpopulations and fibrogenic pathways important for scar formation. Considerable transcriptional heterogeneity was observed within the keratinocyte populations. FAK-deleted keratinocytes demonstrated increased expression of genes integral to mechanotransduction and extracellular matrix production, including Igtbl, Mmpla, and Col4a1. Transcriptional activities upon FAK deletion were not identical across all single keratinocytes, resulting in higher frequency of a minor subpopulation characterized by a matrix-remodeling profile compared to wildtype keratinocyte population. The importance of keratinocyte FAK signaling gene expression was revealed. A minor subpopulation of keratinocytes characterized by a matrix-modulating profile may be a keratinocyte subset important for mechanotransduction and scar formation.

## 1. Introduction

Tissue repair is among the most complex biological processes and occurs through a highly regulated cascade of overlapping biochemical and cellular events [1]. The underlying orchestrators of the wound healing cascade have yet to be fully elucidated [2]. A fundamental paradigm of cutaneous healing in the adult human is that every injury provokes a fibroproliferative response resulting in the formation of a scar [3]. Scar tissue, formed through normal wound healing to re-establish continuity of the integument, represents the “midpoint” in a spectrum of wound healing responses [4]. Aberrations in the process, such as hypertrophic scarring and keloid formation, represent an “over-healing” response, whereas some patients may suffer from “under-healing” in chronic and/or delayed wounds [5]. Both extremes of wound healing can lead to significant functional impairment, psychosocial morbidity, and constitute a significant socioeconomic burden [6].

Although the pathogenesis of over- and under-healing is not completely understood, recent studies have provided significant insight into the pathophysiologic basis of dysfunctional wound healing. Mechanical cues have been identified to play a major role in both chronic wound development as well as hypertrophic scar (HTS) formation [7]. Mechanotransduction pathways influence the homeostasis in virtually all tissues and organ systems [8,9,10]. Mechanical homeostasis in the skin is also a critical regulator of skin biology. For example, the human skin has been shown to have static lines of maximal stress called the Langer’s lines, which surgeons use to orient incisions to minimize scar formation [11]. In addition, joint movement, muscle activity, and gravity can cause dynamic stress to the skin. Mechanical strain introduced by pregnancy, weight gain, or subcutaneously implanted devices can induce an increase in mass, volume, and area of the skin [12]. Disruption of skin homeostasis by wounds, genetic alterations, or diseases can lead to aberrant mechanobiology of the skin [10]. Improved understanding of skin mechanotransduction pathways at baseline levels, under homeostasis and under mechanical stress will clarify skin mechanobiology and its role in normal and pathologic skin disorders.

Recent evidence has linked a central mechanotransduction pathway, i.e., the non-receptor protein tyrosine kinase, focal adhesion kinase (FAK), to both mechanical homeostasis and pro-fibrotic mechanotransduction and other wound healing aberrations [8,9]. Our laboratory was the first to develop and publish the murine model of HTS based on mechanical loading and showed that sustained mechanical forces were able to induce FAK-mediated pro-survival signaling and modify inflammation. In addition, using microarray analysis we have shown in a porcine model and human studies that elevated wound tension caused a pro-fibrotic phenotype and HTS formation. In addition, we recently reported that in diabetic delayed wound healing, FAK degradation by calpain might decelerate wound repair. Integrin-FAK mechanotransduction cascades are involved in fibro-proliferative states such as hepatic fibrosis [13], cardiac hypertrophy [14], vascular smooth muscle atherosclerosis [15], and pulmonary fibrosis [16]. FAK can activate numerous downstream components involved in fibrogenic events such as PI3K/Akt and mitogen-activated protein kinases (MAPK) [17,18,19,20,21,22,23]. How FAK modulates skin cell behavior, especially on other mechanotransduction and wound healing-associated proteins, is not well understood.

Our laboratory has previously demonstrated that FAK is important for mechanotransduction in cutaneous fibroblasts and that a fibroblast-specific deletion of FAK results in reduced fibrosis after injury in a mouse model of scar formation [24]. In contrast, the loss of FAK specifically in keratinocytes leads to significantly delayed wound healing and pathologic dermal proteolysis in mice [25]. Keratinocyte FAK-deleted mice were also found to have decreased dermal thickness and collagen density, findings linked to over-activation of the matrix-remodeling enzyme matrix metalloproteinase 9 (MMP9) [25]. However, a paradoxical upregulation of collagen I and III, the predominant collagen subtypes in cutaneous healing, in these wounds suggests that FAK signaling has a complex effect on extracellular matrix (ECM) repair.

Keratinocytes have been shown to express high levels of collagen based on transcriptome-wide microarray studies [26,27], which suggest a role for these cells in ECM deposition. While global gene expression analyses are sufficient for population wide transcriptional screening, transcriptional profiling of higher resolution is needed to uncover aberrations in cellular signaling only affecting a subset of cells [28]. Since rare, but important subpopulations of cells can significantly alter the biological process of wound healing and mechanical homeostasis, our laboratory has developed a microfluidics-based approach to gene expression analysis that enables the discovery of altered gene expression patterns on a single-cell level [29]. Here we employ this method to evaluate how dysregulation of FAK signaling affects mechanically unstimulated keratinocyte gene transcription at baseline and whether the loss of this mechanical mediator leads to perturbations in cellular subpopulations that may explain the impairments observed at a physiological level.

## 2. Results

### 2.1. Knockout of Keratinocyte FAK (Focal Adhesion Kinase) Alters the Expression of Numerous Genes Integral to Tissue Repair

**Figure 1:**
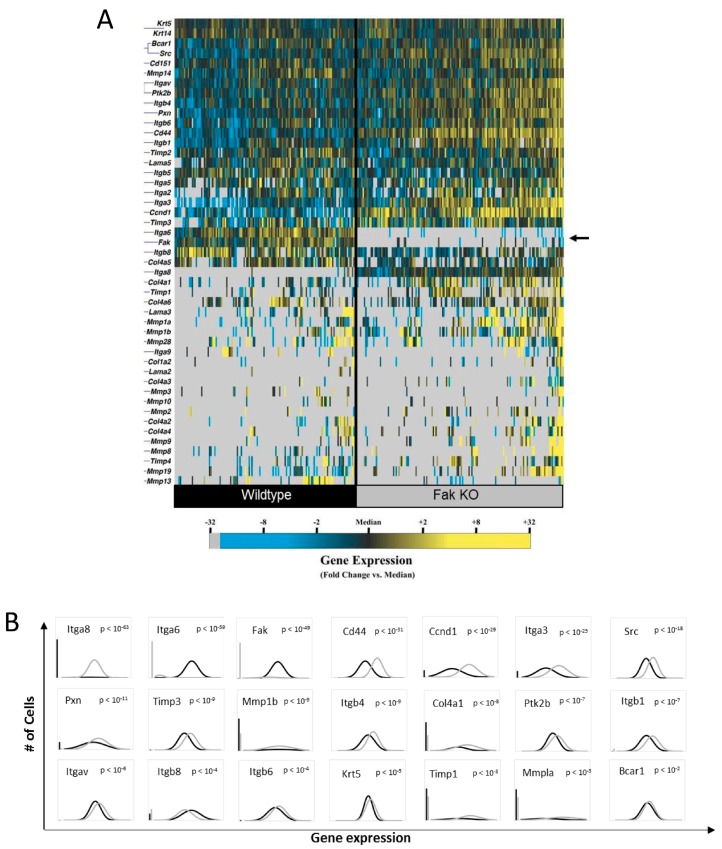
Keratinocyte FAK deletion alters the expression of numerous genes integral to tissue repair. (**A**) Hierarchical clustering of simultaneous gene expression for single cells from WT (left) and FAK KO (right) mice. Gene expression is presented as fold change from median on a color scale from yellow (high expression, 32-fold above median) to blue (low expression, 32-fold below median). Cell/gene qPCR reactions failing to amplify after 40 cycles are designated as non-expressers and represented in gray; (**B**) Differential gene expression between WT and FAK KO cells identified using nonparametric two-sample Kolmogorov-Smirnov testing. Twenty-one genes exhibit significantly different (*p* < 0.01 following Bonferroni correction for multiple comparisons) distributions of single cell expression between populations, illustrated here using median-centered Gaussian curve fits. Black and gray color histogtams denote WT and FAK KO expression, respectively. The left bar for each panel represents the fraction of qPCR reactions that failed to amplify in each group.

Keratinocytes have been shown to influence cutaneous fibrosis and repair [25,30] and are highly mechanoresponsive cells [31,32,33]. Mechanoresponsive cellular components, in particular through activation of FAK, have recently been identified as key mediators in the development of hypertrophic scars, as well as physiological wound healing [24,25]. In an attempt to elucidate how FAK affects gene expression in epithelial cells during cutaneous repair, we compared wildtype (WT) and FAK knockout (KO) keratinocytes (Figure 1) utilizing microfluidic-based single-cell transcriptomics [29]. Single-cell analysis of freshly isolated keratinocytes from WT and keratinocyte-specific FAK-deleted mice demonstrated significant transcriptional heterogeneity both within and across these two groups (Figure 1A,B), with clear lack of any FAK expression in the KO group. Numerous genes were differentially expressed between these cells as a result of FAK deletion (Figure 1B). These include ECM genes such as Collagen type IV (*Col4*) subunits and Keratin 6 (*Krt6*) as well genes that regulate cell-ECM adhesion and ECM-mediated mechanotransduction such as *Fak*, *Cd44*, *Pax*, integrins such as *Itgav* and *Itgb1*, *Itgb4*, *Itgb6*, and *Itgb8*. Factors involved in tissue repair and matrix remodeling such as MMPs and tissue inhibitors of MMPs (TIMPs) were altered in KO cells. For example, expression of *Cd44*, *Itgav*, and *Itgb1* were differentially up-regulated with FAK deletion, suggesting that these signaling regulators are closely associated with FAK and FAK-mediated mechanotransduction network. Interestingly, genes involved in cancer progression including tyrosine protein kinase (*Src*) and breast cancer anti-estrogen resistance protein (*Bcar*) were also altered in FAK KO cells. These data indicate that keratinocyte FAK expression is closely linked to mechanoregulatory factors, as well as mediators implicated in tissue repair and remodeling, underscoring the variety of molecular pathways affected by mechanotransduction component FAK.

### 2.2. FAK-Deleted Keratinocytes Demonstrate Alterations in Key Mechanotransduction and Collagen Signaling Pathways

**Figure 2:**
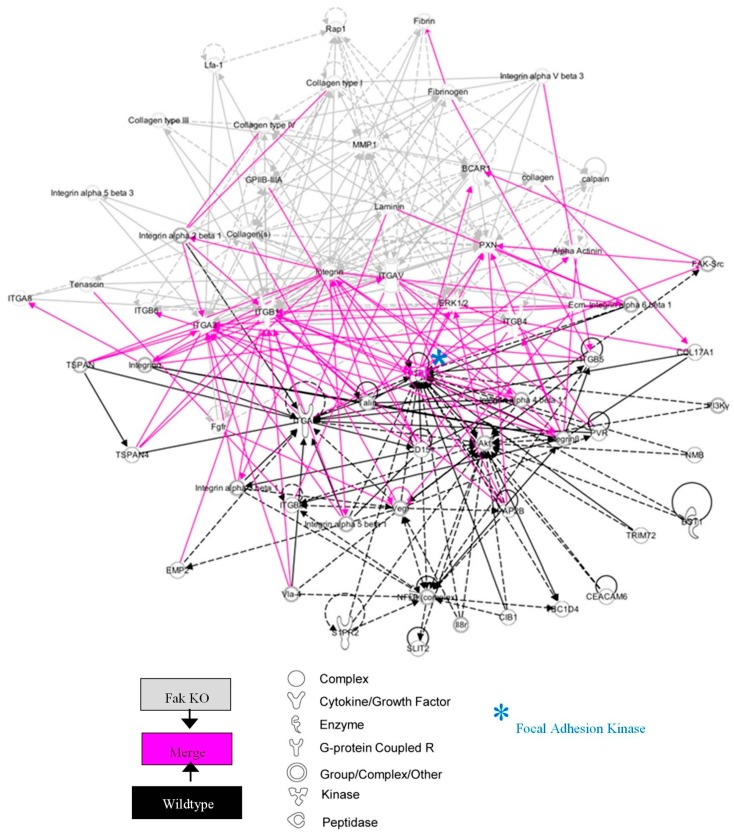
FAK-deleted keratinocytes demonstrate alterations in key mechanotransduction and collagen signaling pathways. The top scoring Ingenuity Pathway Analysis (IPA)-constructed transcriptome network generated from genes significantly up-regulated (grey) and down-regulated (black) in FAK-deleted keratinocytes compared to WT cells were merged using IPA’s Ingenuity Knowledge Base, creating a super-network centered on the FAK-AKT-ERK axis. Direct relationships are indicated by solid lines, and dashed lines represent indirect relationships. Known relationships among molecules across the original two networks are represented in magenta. * denotes FAK (PTK2).

To elucidate the effects of FAK deletion on keratinocyte intracellular signaling, we next identified canonical pathways whose expression was significantly altered using Ingenuity Pathway Analysis (IPA). Analyzing known canonical pathways based on genes up- and down-regulated in FAK-deleted keratinocytes, we found that integrin signaling, FAK signaling, and ERK/MAPK signaling were most highly affected by the loss of keratinocyte FAK. We further utilized IPA to generate transcriptional networks based on over- (Figure S2A) or under- (Figure S2B) expressed genes in the KO cells compared to WT keratinocytes. These included numerous collagen and integrin genes, as well as major upstream regulators such as *Fak*, *Akt*, and *Erk*. When we merged these pathways (Figure 2) we obtained a comprehensive signaling network centered on the FAK-AKT-ERK axis, again demonstrating the critical role of FAK in the regulation of collagen/integrin expression. Notably, an upstream analysis of these differentially expressed genes implicated two key molecular regulators of the transcriptional changes in KO cells. The merged transcriptome network shown in Figure 2 represents features of activation of fibrogenic regulator *Tgfbr2* and suppression of the proto-oncogene *Mycn* that produced the majority of transcriptional changes observed in KO cells.

### 2.3. FAK Deletion Affects Keratinocyte Gene Expression Asymmetrically and Induces a Transcriptionally Activated Subpopulation

**Figure 3:**
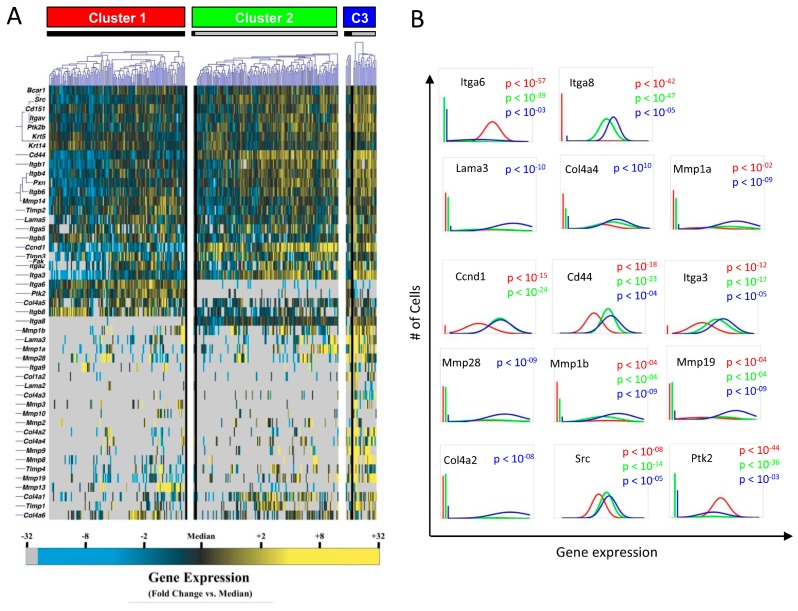
Partitional cluster analysis of single-cell transcriptional data. (**A**) K-means clustering of WT (black bar) and FAK-deleted keratinocytes (grey bar). Gene expression is presented as fold change from median on a color scale from yellow (high expression, 32-fold above median) to blue (low expression, 32-fold below median); (**B**) Differentially-expressed genes among cells in each cluster using non-parametric two sample Kolmogorov-Smimov testing across all (both WT and FAK KO) keratinocytes comprising each cluster, illustrated here using median-centered Gaussian curve fits. The left bar for each panel represents the fraction of qPCR reactions that failed to amplify in each group.

Given the considerable transcriptional heterogeneity observed at the single-cell level and earlier description of keratinocytes as a cell pool composed of distinct subsets [34,35], we applied partitional clustering to identify transcriptionally distinct (and potentially functionally distinct) subpopulations (Figure 3A). We found that keratinocytes could be grouped into three discrete subgroups based on their transcriptional signatures, designated here as clusters 1, 2, and 3. Interestingly, cluster 1 cells were almost exclusively found in WT mice. In contrast, cluster 2 cells were predominantly found in the FAK KO mice. These subgroups were defined by differing expression patterns similar to those of aggregate WT vs KO cells, and the added granularity of this analysis identified additional differentially expressed genes including multiple collagen and MMP targets (Figure 3B). Furthermore, we identified an additional population of cells (cluster 3) that appear to be activated keratinocytes defined by significant overexpression of collagen and MMP transcripts, which were predominantly found in the KO mice. Higher frequency of cluster 3 cells in the KO mice (approximately 80%) suggests that suppression of FAK may trigger activation and proliferation of these cells, thus resulting in the paradoxical over-expression of collagen observed in the wounds of keratinocyte FAK KO mice [25]. As described previously, keratinocyte FAK KO mice demonstrate dermal proteolysis and clinical features of wound chronicity but also over-express certain subtypes of collagen [25]. Identification of cluster 3 keratinocytes in our current analysis provides an explanation for this paradoxical observation [25]. We identified select genes whose transcriptional activities are differential among cells in each cluster across all keratinocytes comprising each cluster. Figure 3B shows multiple differentially-expressed collagen and MMP target genes identified this way. Of interest, expression of integrin α-6 (*Itga6*) and integrin α-8 (*Itga8*) were regulated in an opposite manner in the absence of epithelial FAK. Both integrin subtypes can modulate keratinocyte migration and cell-ECM interactions and are implicated in wound healing. Expression profiles of integrin α-3 (*Itga3*), an integrin subtype involved in epithelial-mesenchymal transition, and *Cd44* cell adhesion molecule were both significantly up-regulated in FAK KO cells [36]. In addition, there was a trend that an array of differentially-expressed MMP proteins was up-regulated with FAK deletion, suggesting that loss of FAK can modulate ECM-remodeling activities and can potentially lead to altered wound healing profiles seen in FAK KO mice.

**Figure 4:**
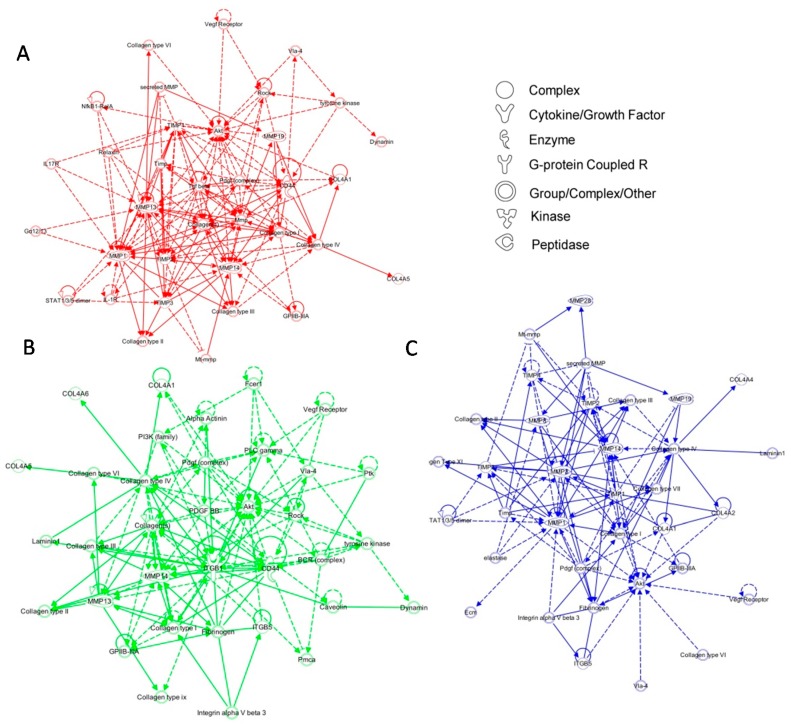
Network analysis of keratinocyte subpopulations. Top scoring Ingenuity Pathway Analysis (IPA)-constructed transcriptome networks based genes that were significantly up-regulated in cluster 1 (**A**; red), cluster 2 (**B**; green), and cluster 3 (**C**; blue). Direct relationships are indicated by solid lines, and dashed lines represent indirect relationships.

**Figure 5:**
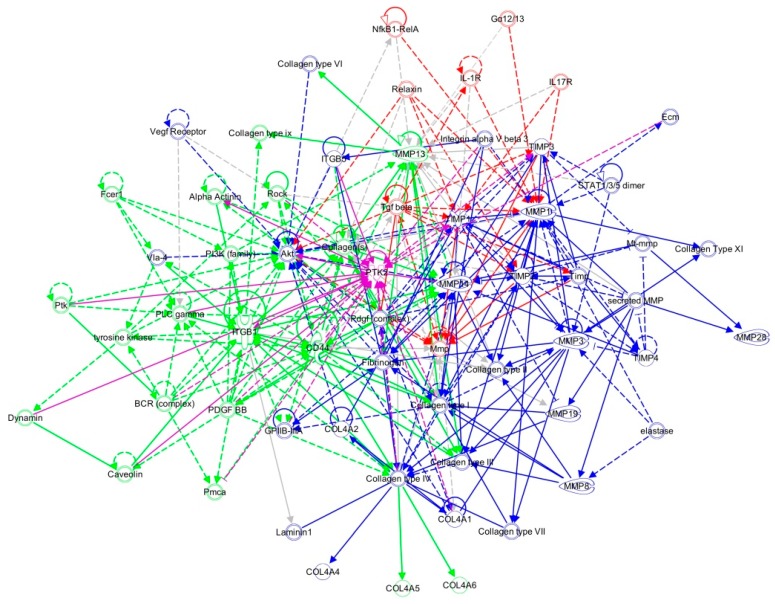
Merged network analysis of keratinocyte subpopulations. Merged network analysis of the three clusters in Figure 4. The top scoring networks generated from genes significantly up-regulated in each of the three keratinocyte subpopulations (cluster 1 (red), cluster 2 (green), and cluster 3 (blue)) were merged using Ingenuity Pathway Analysis (IPA), creating a super-network centered on the FAK-AKT-PI3K axis. Known relationships among molecules across the original three networks are represented in magenta. * denotes FAK (PTK2).

We next applied enrichment analysis to the gene sets up-regulated in cells from each putative subpopulation. Cluster 1 cells were characterized by comparatively increased integrin signaling, FAK signaling, and ERK/MAPK signaling (Figure S3A), consistent with intact FAK expression in these predominantly WT cells. By contrast, cluster 2 cells were associated with ILK signaling and inhibition of MMPs, as well as suppression of paxillin signaling (Figure S3B). Corresponding transcriptional networks were subsequently generated for each subgroup using IPA as described above. Analysis of signaling pathways implicated in cluster 1 resulted in a network defined by increased expression of numerous *Timp* genes (Figure 4A). Of interest, the network of cluster 2 was characterized by elevated expression of various collagen genes (Figure 4B), which was similar to cluster 3 (Figure 4C). Given the complex molecular interactions orchestrated by mechanical signaling [10], we explored intracellular pathways affected by FAK modulation by combining regulatory networks from each keratinocyte subgroup using known relationships among commonly expressed genes (Figure 5). The pathway analysis indicates that a complex signaling cascade centered on the FAK-AKT-P13K axis, may regulate expression of collagen, integrin, and *Mmp* genes. These data suggest that FAK activation/deactivation in keratinocytes has widespread downstream implications and represents a key mechanism underlying the regulation of cellular mechanotransduction, extracellular matrix deposition, and tissue remodeling.

## 3. Discussion

Mechanotransduction pathways have long been shown to regulate human skin biology, scar formation, and wound repair [37]. Previously, our laboratory has identified FAK as a key mechanosensor in both the epidermal and dermal skin compartments with an important role in the cutaneous response to injury [24,25]. Biomechanical aberrations in the wound environment may predispose patients to either hypertrophic scarring or non-healing wounds, both of which are growing public health burdens awaiting effective therapeutics.

In a recent study, we demonstrated that deletion of FAK from epithelial keratinocytes resulted in accelerated dermal proteolysis and delayed wound healing in a mouse model [25]. Here we utilized single-cell microfluidic techniques to identify distinct subgroups of FAK-deleted keratinocytes that may drive this pathologic response. Specifically, one subgroup appears primed to activate matrix degradation and remodeling. Multiple genes linked to wound mechanotransduction were shown to be regulated by FAK, including integrins, *Mmps*, and matrix components, supporting a primary role for epithelial mechanosensing in wound repair. Furthermore, these results suggest that distinct subpopulations of dysfunctional keratinocytes may have the potential to impair normal cutaneous tissue repair.

These findings also confirm the importance of FAK-MAPK intracellular signaling networks in skin mechanobiology [24,25]. The FAK-MAPK pathway has been shown to regulate mechanosensing in numerous tissues throughout the body, including endothelium, bone, heart, and lung [38,39,40,41]. These intracellular pathways may serve as molecular targets to modulate aberrant wound repair. Current device-based approaches to modulate mechanical wound signaling, such as negative pressure wound therapy, are believed to work in part via these intracellular networks [42,43]. An alternative strategy would be to directly target these mechanotransduction pathways pharmacologically, as has been successfully demonstrated in a murine hypertrophic scar model in which small molecule FAK inhibitors were employed [24].

Focal adhesion complexes are dynamic multi-component mechanosensors that influence multiple aspects of cell biology [44,45]. The focus of our study is to investigate the role of epithelial FAK in regulating the transcriptional profiles of mechanically unstimulated keratinocytes at baseline. With loss of epithelial FAK, we observe a corresponding decrease in expression of integrin α-6 (*Itga6*) and increased expression of integrin α-8 (*Itga8*). Integrins are transmembrane receptor complexes that dimerize and transmit extracellular cues (such as mechanical force) into cells. The differential expression and activation of integrins can modify diverse cell behaviors and is highly implicated in wound healing [46]. Itga6 has been closely linked to keratinocyte migration and cell-matrix interactions with the basement membrane component laminin-111 [47]. Itga8 has been less frequently examined in keratinocytes, but studies suggest a role for this protein in epithelial-mesenchymal interactions, matrix remodeling, and cell migration [48,49]. These findings demonstrate that loss of FAK alters the surface integrin profile of keratinocytes and potentially disrupts how the epithelial layer senses the wound environment. It is also interesting to note that expression of *Cd44*, a cell adhesion molecule that has been shown to stimulate activation of FAK via Rho-dependent mechanisms, was up-regulated with loss of epithelial FAK. Since Cd44 has a direct functional link to FAK-mediated signaling and plays a role in suppressing apoptosis, its importance in the context of wound healing may be of future interest. In a similar context, expression of *Ccnd1* was also increased with epithelial FAK deletion, suggesting that cellular proliferation profiles can also be altered with loss of FAK in keratinocytes.

Further, we found that WT keratinocytes demonstrated considerable expression of integrin-FAK-MAPK pathways, whereas FAK-deleted keratinocytes preferentially expressed ILK and suppressed paxillin signaling - known components of focal adhesion complexes [50]. The significance of this is unclear, as both ILK and paxillin activate many of the same targets as FAK. Of interest, increased activation of ILK and dysregulation of paxillin signaling have been linked to pathologic expression of the matrix-degrading enzyme MMP9 [51,52]. This concept of altered biomechanical sensing has also been applied to cancer metastasis, as tumor cells invade adjacent tissues by secreting MMPs and bypassing physical contact inhibition signals. Our current study will form the basis for future studies on characterization of keratinocyte subpopulations under mechanical stimuli.

## 4. Materials and Methods

### 4.1. Animals

All procedures were approved by the Administrative Panel on Laboratory Animal and Care committee (Protocol number APLAC-12080 approved on 21 December 2012) at Stanford University. Wildtype (WT) mice (C57BL/6) were purchased from Jackson Laboratories (Bar Harbor, ME, USA). Keratinocyte-restricted FAK knockout mice were produced as previously described [25]. Transgenic mice were generated by crossing keratinocyte-specific K5-Cre recombinase mice (FVB.Cg-Tg(KRT1-5-cre)5132JIj, purchased from University of North Carolina Mutant Mouse Regional Resource Center) to homozygous floxed FAK mice (B6.129-Ptk2^tm1Lfr^/Mmcd, purchased from University of California Davis Mutant Mouse Regional Resource Center (Davis, CA, USA).

### 4.2. Keratinocyte Harvest and Culture

Primary epithelial keratinocytes were isolated from WT and keratinocyte-specific FAK knockout mice as previously published [53]. Briefly, full thickness skin was harvested from the mouse dorsum using sterile techniques. Specimens were floated in trypsin (Gibco/lnvitrogen, Carlsbad, CA, USA) before adhering the epidermal surface to a culture dish lid. After removing the dermis with forceps, the epidermal specimens were minced and placed into suspension. Cells were grown on plates coated with Coating Matrix Kit (Gibco/lnvitrogen, Waltham, MA, USA) and keratinocyte basal media (Lonza, Walkersville, MD, USA) supplemented with KGM Gold Bullet Kit (Lonza) was used to maintain cells. After a short-term culture for approximately 7 days without subculturing, passage 0 keratinocytes were used for all experiments.

### 4.3. Single-Cell Transcriptional Analysis

Cultured keratinocytes were sorted as single cells into lysis buffer using a Becton Dickinson FACSAria flow cytometer. Live/dead gating was performed based on propidium iodide exclusion. Reverse transcription and low cycle pre-amplification were performed following addition of Superscript Ill reverse transcriptase enzyme (Invitrogen, Waltham, MA, USA), Cells Direct reaction mix (Invitrogen), TaqMan assay primer sets (Applied Biosystems, Foster City, CA, USA) (Figure S1). The resulting cDNA was loaded onto a 48.48 Dynamic Array (Fluidigm, South San Francisco, CA, USA) for qPCR amplification using Universal PCR Master Mix (Applied Biosystems) with TaqMan assay primer sets, and products were analyzed on the BioMark reader system (Fluidigm).

Analysis of single-cell data was performed as previously described [54]. Briefly, expression data from all chips (both WT and FAK KO) were normalized relative to the median expression for each gene in the pooled sample and converted to base 2 logarithms. Absolute bounds of ±5 cycle thresholds from the median were applied, and non-expressers were assigned to this floor. Heatmaps were generated and organized using hierarchical clustering in order to facilitate data visualization using MATLAB (R2011 b, MathWorks, Natick, MA, USA).

Partitional clustering of gene expression data for each group of keratinocytes was performed using a modified k-means algorithm to identify subpopulations based on transcriptional profiles [55]. Gene-wise comparisons between groups and across clusters was achieved using a standard two sample Kolmogorov-Smirnov test with a strict cutoff of *p* < 0.05 following Bonferroni correction for multiple hypothesis testing. For comparisons among subgroups, the empirical distribution of cells from each cluster was evaluated against that of the remaining cells in the experiment.

Canonical pathway calculations and network analyses were performed using Ingenuity Pathway Analysis (IPA, Ingenuity Systems, Redwood City, CA, USA). For these analyses, the gene targets interrogated in the corresponding single-cell analysis (rather than the entire transcriptome) were used as the reference set in order to avoid biasing the associated enrichment and network calculations.

## 5. Conclusions

Our study contributes to improved understanding of FAK-mediated keratinocyte gene transcription by dissecting and highlighting the effects of this mediator and related pathway components in the epidermis. Global suppression of FAK not only restricted to keratinocytes (e.g., using pharmacological FAK inhibitors), however, may result in different pathophysiological outcomes and remains to be studied. In addition, we have shown the existence of a novel subpopulation of keratinocytes that may respond differentially to fibrogenic mechanical cues during the course of wound healing and scar formation. It also remains to be studied how FAK alters the transcriptional profiles of mechanically-strained keratinocytes. The challenge of targeting these cells in vivo to modulate this process remains an ongoing effort, and future studies will clarify the clinical relevance of these single-cell transcriptional findings.

In conclusion, the findings of our study will enhance our understanding of the epidermal mechanotransduction important for scar formation and will have future implications for developing fibrosis and scar therapies targeting FAK-mediated mechanotransduction pathways.

## Supplementary Materials

Supplementary materials can be found at www.mdpi.com/1422-0067/18/9/1915/s1.
