# Supplementary material for: The Role of Focal Adhesion Kinase in Keratinocyte Fibrogenic Gene Expression
Source: Int J Mol Sci. 2017 Sep 7;18(9):1915. doi: 10.3390/ijms18091915 (PMC5618564; doi:10.3390/ijms18091915)
Supplement: Supplementary file 1 [file ijms-18-01915-s001.pdf]

Gene ID	Assay ID	Gene ID	Assay ID
Itgb1	Mm01253230_m1	*Col4a3*	Mm00483656_m1
Itga2	Mm00434371_m1	*Col4a4*	Mm00801574_m1
Itga3	Mm00442890_m1	*Col4a5*	Mm00801606_m1
Itga5	Mm00439797_m1	*Col4a6*	Mm00474735_m1
Itga6	Mm00434375_m1	*Lama3*	Mm01254735_m1
Itga8	Mm01324958_m1	*Lama5*	Mm01222029_m1
Itga9	Mm01348480_m1	*Lama2*	Mm00550083_m1
Itgav	Mm00434506_m1	*Mmp1a*	Mm00473485_m1
Itgb4	Mm01266840_m1	*Mmp1b*	Mm00473493_g1
Itgb5	Mm00439825_m1	*Mmp2*	Mm00439498_m1
Itgb6	Mm01269869_m1	*Mmp3*	Mm00440295_m1
Itgb8	Mm00623991_m1	*Mmp8*	Mm00439509_m1
Actb	Mm01205647_g1	*Mmp9*	Mm00442991_m1
Ccnd1	Mm00432359_m1	*Mmp10*	Mm01168399_m1
Krt5	Mm00503549_m1	*Mmp13*	Mm00439491_m1
Krt14	Mm00516876_m1	*Mmp14*	Mm00485054_m1
Col1a2	Mm01165187_m1	*Mmp19*	Mm00491300_m1
Fak	Mm00433205_m1	*Mmp28*	Mm00712992_m1
Ptk2b	Mm00552827_m1	*Timp1*	Mm00441818_m1
Bcar1	Mm00487210_m1	*Timp2*	Mm00441825_m1
Src	Mm00436785_m1	*Timp4*	Mm00446568_m1
Pxn	Mm00448533_m1	*Timp3*	Mm00441826_m1
Cd151	Mm00515411_m1	*Cd44*	Mm01277163_m1
Col4a1	Mm01210125_m1	*Col4a3*	Mm00483656_m1

Figure S1. TaqMan assay primer sets. TaqMan assays used to interrogate gene expression within murine keratinocytes. All assays were obtained from Applied Biosystems (Foster City, CA).

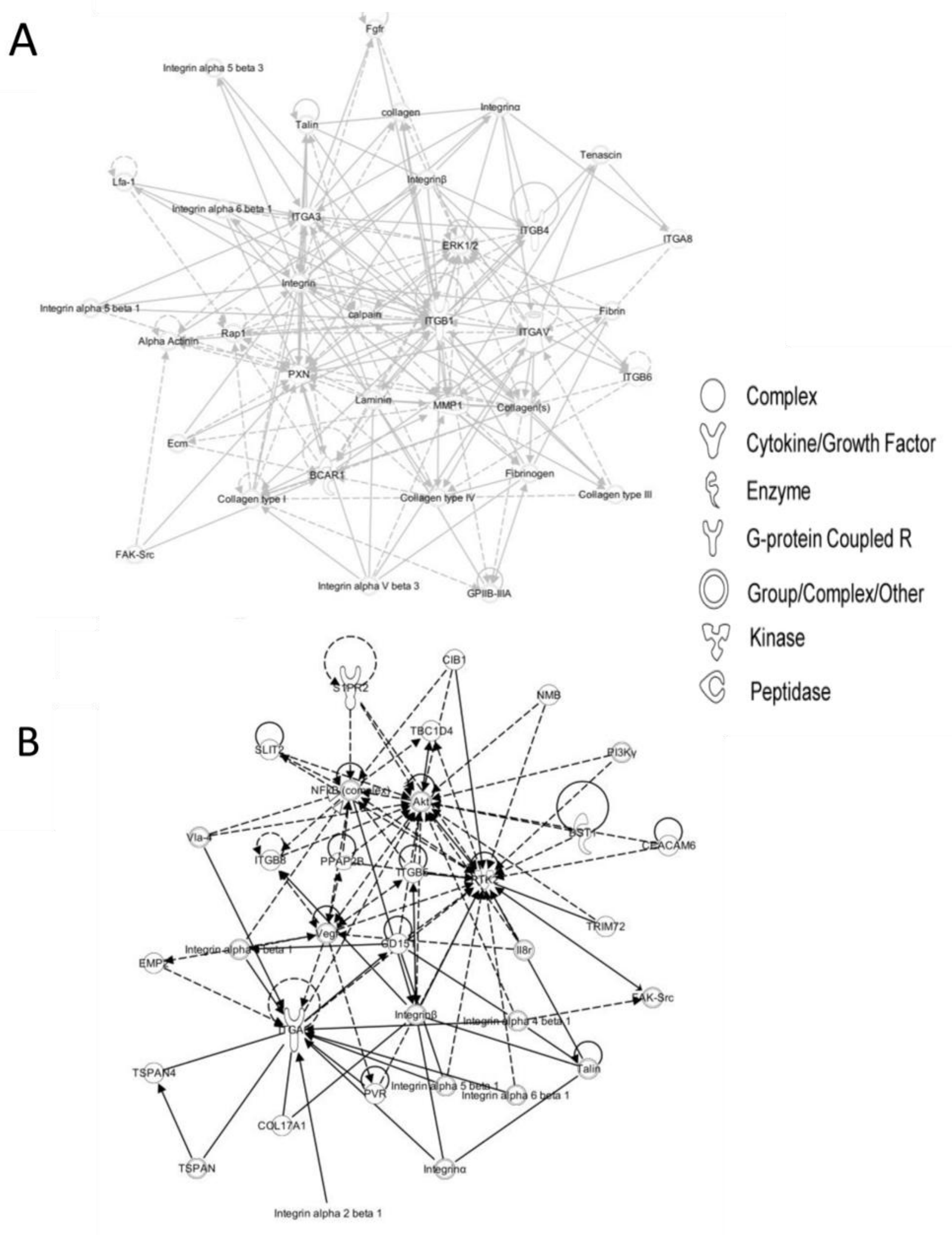

Figure S2. Merged network analysis of wildtype and FAK-deleted keratinocytes. Top scoring Ingenuity Pathway Analysis (IPA)-constructed transcriptome networks based genes that were significantly up-regulated (**A**) or down-regulated (**B**) in FAK-deleted keratinocytes compared to wildtype cells. Direct relationships are indicated by solid lines, and dashed lines represent indirect relationships.

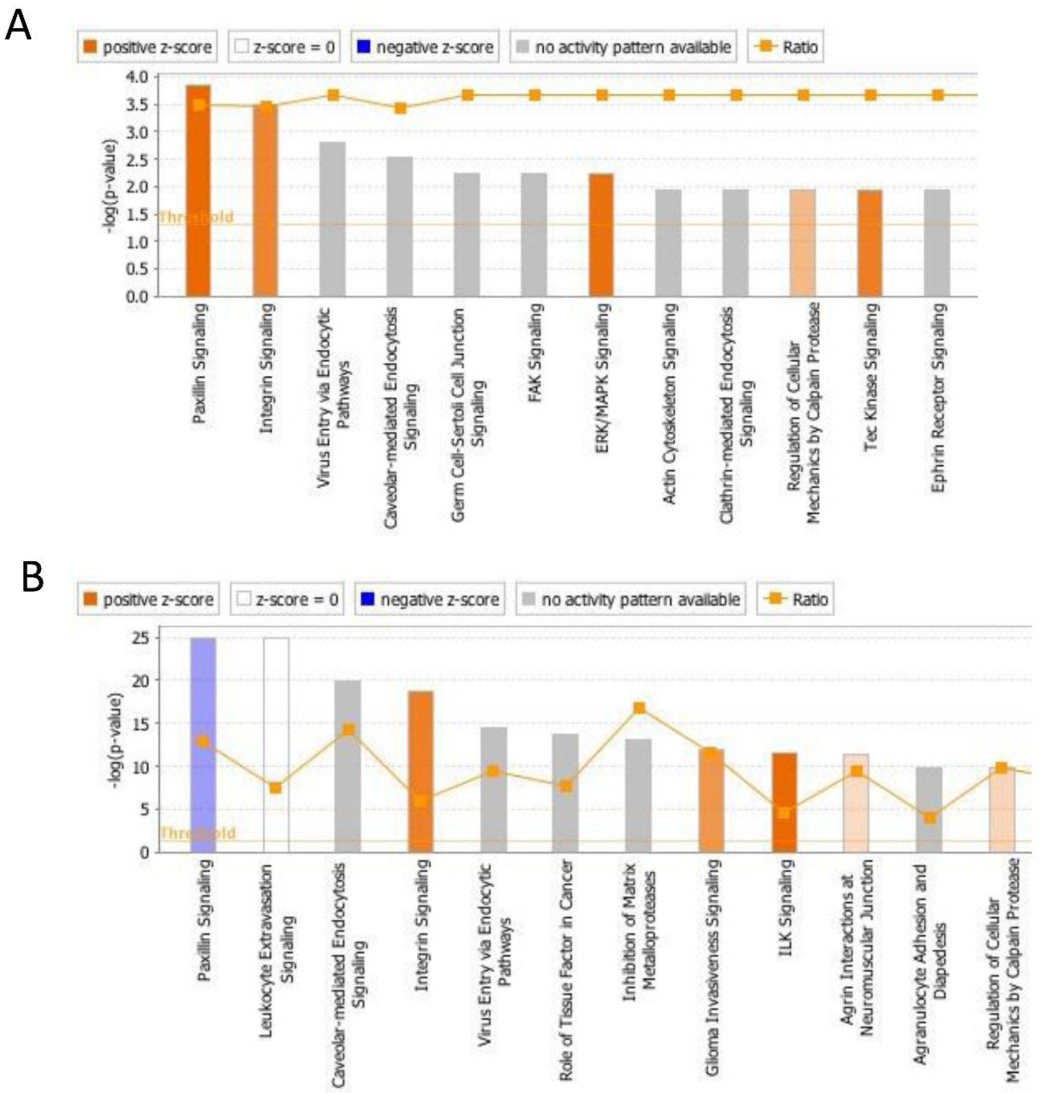

Figure S3. Canonical pathway analysis of keratinocyte subpopulations. Canonical pathways significantly enriched for among genes whose expression was significantly up-regulated in cluster 1 (**A**) and cluster 2 (**B**) cells based on the partitions delineated in Figure 3.
